# Feasibility and Clinical Outcomes of a Step Up Noninvasive Respiratory Support Strategy in Patients with Severe COVID-19 Pneumonia

**DOI:** 10.3390/jcm10225444

**Published:** 2021-11-22

**Authors:** Silvia Coppola, Pierachille Santus, Giovanni Sotgiu, Michele Mondoni, Alessia Gandola, Marina Saad, Giuseppe Francesco Sferrazza Papa, Stefano Centanni, Laura Saderi, Davide Alberto Chiumello, Dejan Radovanovic

**Affiliations:** 1 Department of Anesthesia and Intensive Care, San Paolo Hospital, ASST Santi Paolo e Carlo, Via di Rudini 8, 20142 Milano, Italy; silvia_coppola@libero.it (S.C.); alessia.gandola@asst-santipaolocarlo.it (A.G.); 2 Department of Biomedical and Clinical Sciences (DIBIC), Università degli Studi di Milano, Via G.B. Grassi 74, 20157 Milano, Italy; pierachille.santus@unimi.it (P.S.); marina.saad@unimi.it (M.S.); 3 Division of Respiratory Diseases, L. Sacco University Hospital, ASST Fatebenefratelli-Sacco, Via G.B. Grassi 74, 20157 Milano, Italy; dejan.radovanovic@asst-fbf-sacco.it; 4 Dipartimento di Scienze Mediche, Chirurgiche e Sperimentali, Università degli Studi di Sassari, Viale San Pietro, 07100 Sassari, Italy; gsotgiu@uniss.it (G.S.); lsaderi@uniss.it (L.S.); 5 Respiratory Unit, San Paolo Hospital, ASST Santi Paolo e Carlo, Via di Rudinì 8, 20142 Milano, Italy; michele.mondoni@asst-santipaolocarlo.it (M.M.); stefano.centanni@unimi.it (S.C.); 6 Department of Health Sciences, Università degli Studi di Milano, Via di Rudinì 8, 20142 Milano, Italy; giuseppe.sferrazza@unimi.it; 7 Department of Neurorehabilitation Sciences, Casa di Cura del Policlinico, Via Giuseppe Dezza 48, 20144 Milano, Italy; 8 Coordinated Research Center on Respiratory Failure, Università degli Studi di Milano, Via di Rudini 8, 20142 Milano, Italy

**Keywords:** COVID-19, noninvasive ventilation, continuous positive airway pressure, intubation, mortality, acute respiratory failure

## Abstract

The best noninvasive respiratory strategy in patients with Coronavirus Disease 2019 (COVID-19) pneumonia is still discussed. We aimed at assessing the rate of endotracheal intubation (ETI) in patients treated with continuous positive airway pressure (CPAP) and noninvasive ventilation (NIV) if CPAP failed. Secondary outcomes were in-hospital mortality and in-hospital length of stay (LOS). A retrospective, observational, multicenter study was conducted in intermediate-high dependency respiratory units of two Italian university hospitals. Consecutive patients with COVID-19 treated with CPAP were enrolled. Thoraco-abdominal asynchrony or hemodynamic instability led to ETI. Patients showing SpO_2_ ≤ 94%, respiratory rate ≥ 30 bpm or accessory muscle activation on CPAP received NIV. Respiratory distress and desaturation despite NIV eventually led to ETI. 156 patients were included. The overall rate of ETI was 30%, mortality 18% and median LOS 24 (17–32) days. Among patients that failed CPAP (*n* = 63), 28% were intubated, while the remaining 72% received NIV, of which 65% were intubated. Patients intubated after CPAP showed lower baseline PaO_2_/FiO_2_, lower lymphocyte counts and higher D-dimer values compared with patients intubated after CPAP + NIV. Mortality was 22% with CPAP + ETI, and 20% with CPAP + NIV + ETI. In the case of CPAP failure, a NIV trial appears feasible, does not deteriorate respiratory status and may reduce the need for ETI in COVID-19 patients.

## 1. Introduction

Coronavirus disease 2019 (COVID-19) is an infectious disease caused by a new pathogen named severe acute respiratory syndrome coronavirus 2 (SARS-CoV-2). Acute respiratory failure and acute respiratory distress syndrome (ARDS) caused by bilateral interstitial pneumonia are some of the most severe complications of COVID-19 [1,2]. Previous studies showed that up to 20% of patients need hospital admission [3], with an in-hospital mortality ranging from 16% to 78% [1,2,4,5]. Patients with respiratory failure frequently experience hypoxemia, increased respiratory rate and inspiratory effort [6]. Moreover, differently from typical ARDS, the pathophysiology of COVID-19-related ARDS is characterized by different degrees of micro/macro-thrombosis and by regional dysregulation of lung blood flow [7,8,9], which contribute to the ventilation-perfusion mismatch and increased shunt fraction [8,9]. Respiratory support should reduce the inspiratory effort and the pulmonary stress (i.e., patient self-inflicted lung injury) [10,11,12]. Based on the severity of acute respiratory failure, the respiratory support can include high flow oxygen therapy, continuous positive airway pressure (CPAP), noninvasive (NIV) and invasive mechanical ventilation (IMV) [6]. Early European consensus statements for the management of non-critically ill COVID-19 patients with acute respiratory failure recommended Helmet CPAP as first choice, the mask CPAP as the second choice and NIV applied with face mask as last option [3,13,14]. On the other hand, the Italian Society of Anti-Infective Therapy and Italian Respiratory Society suggested that Helmet CPAP should be the first line of respiratory support with a PEEP titrated not exceeding 12 cm H_2_O based on a patient’s needs, tolerability and adverse events [15,16]. Conversely, the Surviving Sepsis Campaign did not make any recommendations regarding the use of CPAP, providing only a weak recommendation for NIV [17].

The proportion of patients treated with noninvasive respiratory supports may vary from 62% in China to 20% and 11% in North America and Italy, respectively [18,19]. Mortality rate does not differ in patients initially treated with a noninvasive respiratory support and subsequently intubated in comparison with those immediately treated with IMV when admitted to hospital [2].

However, the majority of the studies were performed in intensive care units and only several data are available for patients treated with CPAP and/or NIV outside the intensive care units [19,20,21,22,23,24,25,26]. In the latter case, helmet CPAP is usually prescribed [19,21], with a failure rate ranging from 27% to 44% and a mortality rate from 25% to 30% [19,20,21,26].

CPAP failure (i.e., persistent severe hypoxemia or high respiratory rate and inspiratory effort) could be followed in selected cases by a NIV trial before implementing IMV [27]. However, noninvasive respiratory support in patients with very severe respiratory failure may favor a delayed intubation, increasing mortality [28]. IMV should be promptly provided in the case of deterioration of the clinical conditions [29]. A large Italian retrospective study recently showed that patients treated with helmet CPAP or NIV had comparable outcomes [20]; however, patients failing CPAP were directly intubated without a NIV trial.

The aim of the present study was to retrospectively assess the intubation rate of a noninvasive respiratory strategy based primarily on the prescription of helmet CPAP and NIV in the case of CPAP failure, in COVID-19 patients treated in non-intensive care settings.

## 2. Materials and Methods

### 2.1. Study Population

Adults (>18 years) with acute respiratory failure caused by COVID-19 pneumonia, laboratory-confirmed SARS-CoV-2 infection, with ground glass bilateral opacities at chest X-ray or lung CT were consecutively enrolled. They were admitted to the intermediate-high dependency respiratory units of two University Hospitals in Milano (Italy) between March and May 2020.

They were included if eligible for CPAP [21,30]. As suggested by previous reports [21,30], inclusion criteria were: PaO_2_/FiO_2_ ratio <300 and/or dyspnea, tachypnea (respiratory rate > 30 bpm) or activation of respiratory accessory muscles while on Venturi or Reservoir mask delivering up to 12 L/min (FiO_2_ of at least 0.5). Exclusion criteria were: the need for immediate endotracheal intubation (ETI), unstable hemodynamics, delirium, Glasgow coma scale < 15 and respiratory acidosis. Only a very limited percentage of patients with COVID-19 pneumonia present with hypercapnia at admission [21]. Decompensated hypercapnic respiratory failure at admission, if eligible, was considered a criterion for direct intubation, while patients with a history of nocturnal hypoventilation or obstructive sleep apnea with chronic hypercapnia were not excluded from the study. Patients receiving a “do not intubate” (DNI) order, thus not eligible to ETI and with noninvasive respiratory support as the ceiling treatment, were excluded from the study. A DNI order was determined after a multidisciplinary discussion among the high dependency respiratory unit and critical care unit staff and shared with the patient and the family, and was based upon survival chances, comorbidities, clinical status, frailty and, when possible, the patient’s decision. The study was approved by the local ethical committee (Comitato Etico Milano Area I; 17263/2020–2020/ST/095).

### 2.2. Study Design

CPAP was delivered through a high flow generator (VitalSigns Inc., Totowa, NJ, USA; 90–140 L/min; Myo 3133 A, Pulmodyne, Indianapolis, IN, USA), using a helmet (Starmed, Teramo, Italy) with a mechanical PEEP valve. The PEEP levels ranged between 7.5 and 10 cm H_2_O and FiO_2_ was titrated to maintain a SpO_2_ > 94%. CPAP failure was defined by the presence of tachypnea (respiratory rate > 30 bpm), accessory muscle activation, oxygenation worsening, poor tolerability to the device despite adequate sedation or if the patient developed respiratory acidosis or alkalosis. In the case of CPAP failure, a NIV trial was implemented, with the only exception of patients with severe respiratory distress (i.e., activation of neck, pectoralis, abdomen, transverse and intercostal muscles, respiratory asynchrony with/without agitation or delirium) where ETI was performed and IMV started. NIV was delivered by a mechanical ventilator (MONNAL T60, Air Liquide Medical Systems, Antony, France) with a facial or oro-nasal mask (Armstrong Medical, Coleraine, UK). The level of PSV during NIV was set between 10–12 cm H_2_O and subsequently adjusted to achieve an acceptable tolerability profile and a comfortable respiratory rate. PEEP and FiO_2_ were titrated as well as during CPAP. In the case of NIV failure patients were intubated and started on IMV. Criteria for ETI during NIV were the same adopted for CPAP failure.

### 2.3. Data Collection

Demographics, comorbidities and chronic therapies were recorded at admission. Respiratory rate, gas exchange-related variables, laboratory parameters and ventilatory settings were collected at the emergency department and at the time of CPAP or NIV failure. Blood gas analysis, vital signs, respiratory mechanics and occurrence of respiratory distress were evaluated day by day. Patients’ data were recorded daily up to CPAP or NIV failure or until weaning from CPAP or NIV was started. Weaning criteria were: PaO_2_/FiO_2_ > 200 mmHg, respiratory rate < 20–22/min and PaCO_2_ > 35 mmHg and “weaned” condition achieved when the noninvasive respiratory support was not administered for >24 h.

### 2.4. Outcomes

The primary outcome was the failure rate of noninvasive respiratory support (need for ETI), whereas the secondary outcomes were the in-hospital length of stay and mortality.

### 2.5. Statistical Analysis

Qualitative variables were described with absolute and relative (percentage) frequencies. Quantitative variables were summarized with means (standard deviations, SD) or medians (interquartile ranges, IQR) depending on their parametric or non-parametric distribution, respectively. A chi-squared or Fisher exact test was used to statistically compare qualitative variables. Student’s t or Mann-Whitney test was used to assess statistically significant differences related to parametric and non-parametric variables, respectively. Kaplan Meier curves were plotted to assess differences in terms of main outcomes (e.g., mortality) between groups that were intubated after CPAP and patients that underwent a NIV trial; a long-rank test was used to evaluate the statistical significance. A two-tailed *p*-value less than 0.05 was considered statistically significant. The statistical software STATA version 16 (StataCorp LLC, College Station, TX, USA) was used to perform all statistical computations.

## 3. Results

**Figure 1:**
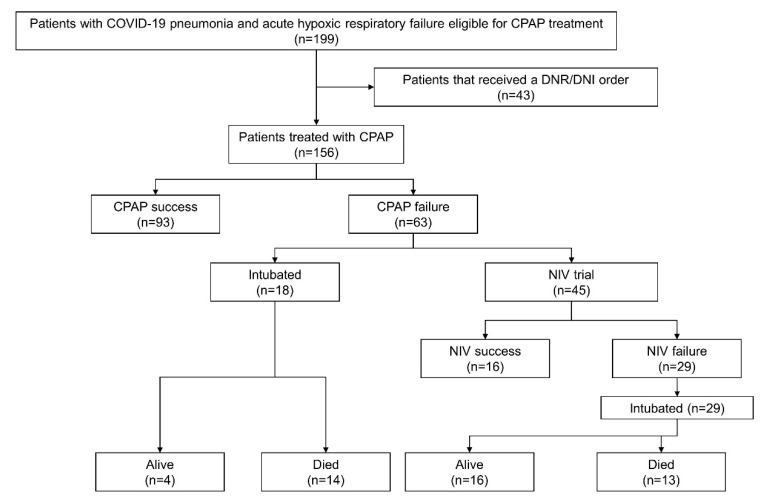
A schematic overview of the studied population. CPAP = continuous positive airway pressure; NIV = noninvasive ventilation; DNR = “do not resuscitate” order; DNI = “do not intubate” order.

A total of 199 patients were evaluated and 156 were recruited (Figure 1). The median (IQR) age of the cohort was 61 (55–69) years, with 76% males, 37% with hypertension, 11% with ischemic heart disease and 16% with diabetes (Table 1). During the admission median (IQR) PaO_2_/FiO_2_ ratio, respiratory rate and arterial carbon dioxide (PaCO_2_) were 269 (168–310), 24 (22–27) bpm, and 33 (30–38) mmHg, respectively. The median (IQR) length of hospital stay was 24 (17–32) days.

Forty-seven (30%) patients showed a noninvasive respiratory support (both CPAP and CPAP + NIV) failure and were intubated with an overall in-hospital mortality of 18%. No patient died after being weaned from CPAP or CPAP-NIV (Figure 1).

### 3.1. CPAP: Success vs. Failure

In patients exposed to helmet CPAP (*n* = 156) CPAP was prescribed for a median (IQR) time of 4 (2–7) days, with a median PEEP of 10 (10–10) cm H_2_O. Ninety-three (60%) patients with CPAP were successfully treated (CPAP success group) without any other respiratory support, and 63 (40%) failed (CPAP failure group) (Figure 1).

**Figure 2:**
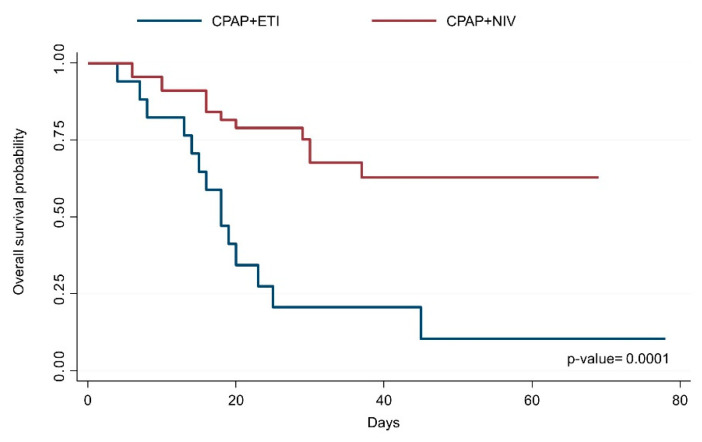
Probability of survival after CPAP failure in patients directly intubated and patients treated with NIV. CPAP = continuous positive airway pressure; NIV = noninvasive ventilation.

In the CPAP failure group, 18 patients (28%) were intubated (CPAP + ETI group) and 45 (72%) received NIV (CPAP + NIV group). Mortality was 22% (14/63) after CPAP + ETI and 20% (13/63) after CPAP + NIV (Figure 2).

When admitted to hospital, CPAP success and failure groups showed similar median (IQR) PaO_2_/FiO_2_ (267 (169–312) vs. 271 (151–295) mmHg; *p*-value = 0.52), respiratory rate (24 (22–27) vs. 24 (22–28) bpm; *p*-value = 0.38) and PaO_2_ (33 (30.1–39.9) vs. 33 (30.3–35.3) mmHg; *p*-value = 0.30) (Table 1).

Patients who failed CPAP had a shorter median (IQR) CPAP duration (2 (1–4) vs. 6 (4–9) days; *p*-value < 0.0001) and a higher in-hospital mortality (42.9%, 27/63, vs. 0.0%, 0/93, *p*-value < 0.001), whereas the median (IQR) length of hospital stay was comparable 23 (17–30) vs. 25 (17–38) days; *p*-value = 0.41) (Table 1).

Considering the CPAP failure group, at the hospital admission the 18 patients directly intubated after CPAP failure (CPAP + ETI group) compared with the 45 patients treated with NIV (CPAP + NIV group) had lower median (IQR) PaO_2_/FiO_2_ ratio (151 (91–267) vs. 281 (209–321) mmHg; *p*-value = 0.005) and higher median (IQR) PaCO_2_ (35 (33–40) vs. 32 (29–35) mmHg; *p*-value = 0.002), with a similar median (IQR) respiratory rate (25 (24–30) vs. 24 (22–28) bpm; *p*-value = 0.674). The CPAP + ETI group showed lower lymphocyte counts, neutrophil percentage, higher values of INR, LDH, D-dimer and bilirubin when compared with patients treated with CPAP + NIV (Table 2).

At time of CPAP failure, the median (IQR) PaO_2_/FiO_2_ ratio of the CPAP + ETI group was significantly lower (99 (82–112) vs. 143 (121–190) mmHg; *p*-value < 0.001) and mean (SD) respiratory rate was significantly higher (35 (9) vs. 25 (6.5) bpm; *p*-value < 0.0001) when compared with the CPAP + NIV group.

The median (IQR) duration of CPAP tended to be higher in the CPAP + ETI group (2.5 (2–7) vs. 2 (1–3) days; *p*-value = 0.05) with equal median (IQR) PEEP levels (10 (10–10) vs. 10 (10–10) mmHg; *p*-value = 0.682) (Table 2) compared with the CPAP + NIV group.

### 3.2. CPAP + NIV: Success vs. Failure

Among the 45 patients treated with CPAP + NIV, 29 (64%) failed NIV and were intubated (CPAP + NIV + ETI), whereas 16 (36%) were not intubated.

When admitted to hospital PaO_2_/FiO_2_, respiratory rate and PaCO_2_ were not different. The median (IQR) duration of CPAP before NIV was not different (2 (1.0–3.5) in CPAP + NIV vs. 2 (1–3) days in CPAP + NIV + ETI), whereas median (IQR) NIV duration was significantly lower in patients that were intubated (1 (1–2) vs. 5 (2.5–5) days).

A similar median (IQR) hospital length of stay was recorded (28.5 (24.5–36.5) vs. 29 (17–39) days), whereas in-hospital mortality was significantly higher in patients who failed NIV (45% (13/29) vs. 0% (0/16)) (Table 3).

### 3.3. Intubated Patients: CPAP vs. CPAP + NIV

The overall mortality of intubated patients both after CPAP and after CPAP + NIV failure was 57% (27/47).

Comparing intubated patients that failed CPAP (*n* = 18) and CPAP + NIV (*n* = 29), the mortality was higher in the CPAP + ETI group (77% (14/18) vs. 45% (13/29)). The median (IQR) time spent on CPAP compared with the total time spent on CPAP + NIV was not different (2.5 (2–5) vs. 4 (3–5) days). At the admission, the CPAP + ETI group showed a lower PaO_2_/FiO_2_, lower lymphocyte counts and higher values of INR, LDH, D-dimer and bilirubin, whereas, at the time of intubation, PaO_2_/FiO_2_, respiratory rate and PaCO_2_ were similar (Table 4).

## 4. Discussion

The main findings of the present retrospective study on COVID-19 patients treated with CPAP as first choice or with NIV after the failure of CPAP can be summarized as follows: (1) the overall intubation rate was 30%, 28% of patients that failed CPAP and 64% of patients that failed both CPAP and the subsequent NIV trial, (2) NIV avoided intubation in 35% of patients that failed CPAP; (3) overall in-hospital mortality was 18%: in patients that failed CPAP and were intubated mortality was 22%, while it was 20% in those treated with a NIV trial, and (4) length of hospital stay was similar in patients that succeeded or failed CPAP or NIV.

Hospitalized patients with COVID-19 pneumonia show acute hypoxemic respiratory failure caused by a diffuse alveolar/vascular damage and dyspnea. Oxygen therapy is the first therapeutic approach, with a target of arterial saturation between 92–96% [17]. However, in the case of persistent hypoxemia, increased respiratory rate and dyspnea, a noninvasive respiratory support should be prescribed. The easiest noninvasive respiratory support is CPAP with mask or helmet [31]. The CPAP, by applying a PEEP, should increase the alveolar recruitment, reduce the work of breathing, and improve oxygenation 6. The helmet and face mask reduce the inspiratory effort during continuous flow CPAP [32]. However, the helmet CPAP is usually better tolerated, and should be chosen in the case of long-term exposure [31]. In non-COVID-19 acute respiratory failure, CPAP improves oxygenation, reduces the need of intubation and the risk of intensive care [33,34]. A recent systematic review showed that helmet CPAP was superior to face mask in reducing the rate of ETI and mortality [35]. In COVID-19 patients with acute respiratory failure, several European consensus documents recommend CPAP, administered by helmet both due to the higher number of patients treated in non-intensive care settings and to reduce the risk of environmental spread of aerosols. The helmet requires only a high flow oxygen—air source without necessitating electricity and allowing the patients to be fed and hydrated orally [6]. Recent studies showed the effective prolonged prescription of noninvasive respiratory supports in intensive and non-intensive care settings [1,2,3,4,5,13,14,15,16,17,18,19,20,21,22,23,24,25,26].

However, a protracted use of a noninvasive respiratory support not associated with a clinical recovery can increase the risk of mortality compared with an early adoption of IMV [28]. Thus, a decisional and monitoring algorithm for noninvasive respiratory support should reduce the number of failed patients [30]. An Italian study on noninvasive respiratory support outside the intensive care found that 85% were treated with CPAP with 68% using the helmet. The rate of failure in terms of intubation rate was 37% [19]. Aliberti et al. found a failure rate of 44% [21].

The comparison was difficult due to heterogeneous settings, patients and protocols for noninvasive respiratory support; the ETI rate was slightly lower (30%) in our population. Our results are in line with the multicenter observational study by Franco et al., who found failure rates of 29% and 25% for CPAP and NIV, respectively [20].

The mortality rate of previous studies ranged from 25% to 30% [19,20,21,26]. The overall mortality was low (18%) in our study, with the highest chance of survival for patients that continued to be exposed to a noninvasive respiratory support in comparison with those that failed after CPAP or after CPAP + NIV and were intubated. Similarly, Grasselli et al. found that patients treated noninvasively and subsequently intubated had a significantly lower survival compared with those who continued to receive noninvasive support [2]. When NIV is successful it might significantly decrease mortality [12].

Following our noninvasive respiratory strategy, 40% of patients failed CPAP and 72% of them continued the noninvasive ventilatory support with NIV (i.e., CPAP + NIV group). Patients directly intubated after CPAP had a more severe disease when admitted to hospital, with lower PaO_2_/FiO_2_, higher PaCO_2_, lower lymphocyte counts and higher levels of D-Dimer when compared with the CPAP + NIV group [36]. Accordingly, at the time of failure, after two days of CPAP treatment, intubated patients had a significantly lower PaO_2_/FiO_2_ and higher respiratory rate. Severity of pneumonia according to American Thoracic Society and Infectious Diseases Society of America (ATS/IDSA) criteria [37], higher values of IL-6 and lower platelet counts are risk factors for noninvasive support failure [19,21].

Our clinical strategy was designed to provide a NIV trial to the subgroup of patients showing initial signs of recruitment of the accessory respiratory muscles while on CPAP and were not directly intubated. So far, NIV has been prescribed to treat acute hypoxemic respiratory failure through oro-nasal, full face and helmet devices [30]. NIV could decrease the inspiratory effort and dyspnea better than CPAP, avoiding intubation [30]. However, by applying a pressure support during inspiration, NIV could increase the transpulmonary pressure and tidal volume (i.e., promote the PSILI) and delay the initiation of mechanical ventilation [27,28]. To the best of our knowledge, this is the first study that proposed a step-up strategy in terms of noninvasive respiratory support (i.e., from oxygen supplementation to CPAP and NIV), integrating respiratory failure parameters and clinical criteria to determine the timing of escalation.

In our cohort, 35% who received NIV avoided intubation. Overall, patients received the NIV support for a median of 5 days, but patients that failed were treated for a shorter period in comparison with those who were weaned from NIV. Furthermore, the mortality of intubated patients was significantly higher after CPAP failure than after CPAP + NIV failure (77% vs. 45%), potentially caused by a more severe disease at hospital admission, characterized by lower lymphocytes and neutrophil percentage, higher values of INR, LDH, D-dimer and bilirubin. Indeed, maintaining patients on spontaneous breathing during NIV did not increase the mortality if compared with an early initiation of invasive mechanical ventilation, although it could increase the risk of self-inflicted lung injury. This is in accordance with the results of a recent systematic review, that included more than 8000 patients critically ill patients with COVID-19 pneumonia and demonstrated that timing of intubation may have no effect on in-hospital mortality, suggesting the possibility for a positive role of a “wait and see” approach [38]. Of note is that none of the patients receiving CPAP or CPAP + NIV were intubated in severe distress or hemodynamic instability after failure.

Several study limitations can be found. This is a retrospective study, which did not objectively assess the inspiratory effort using an esophageal catheter or by ultrasound. Data on prone position were not collected and thus the possible effect on clinical outcomes could not be assessed. Furthermore, the small sample size can affect the inference of the findings, although the study sample in terms of age and male prevalence was comparable with previous reports, both including patients admitted in the ICU [39] and in high dependency respiratory units [21].

## 5. Conclusions

In conclusion, the majority of COVID-19 patients with acute hypoxemic respiratory failure can be managed with noninvasive respiratory support without the need for immediate ETI. A noninvasive respiratory support strategy is also feasible outside ICU, provided strict daily monitoring. For the first time we demonstrated that in the case of CPAP failure, a NIV trial can avoid intubation and does not seem to increase mortality and deteriorate patients’ respiratory status. However, a subset of patients who needed direct intubation after CPAP failure for severe respiratory distress is characterized by worse clinical outcomes probably due to faster and severe evolution of disease.

The present noninvasive respiratory strategy needs further validation in larger prospective studies.

## Figures and Tables

**Table 1:** Clinical characteristics of the study cohort and of patients that failed and succeeded CPAP.

		Total (*n* = 156)	CPAP Success (*n* = 93)	CPAP Failure (*n*= 63)	*p*-Value
Males, *n* (%)		119 (76.3)	65 (69.9)	54 (85.7)	**0.02**
Age, years		61 (55.5–69)	63 (56–71)	59 (55–65)	**0.01**
Days from symptoms onset to hospital admission		8 (6–11)	8 (7–11)	8 (6.0–10.5)	0.06
Previous respiratory disease, *n* (%)		11 (7.1)	6 (6.5)	5 (7.9)	0.76
**Comorbidities**					
Smoke, *n* (%)		19/152 (12.5)	16/90 (17.8)	3/62 (4.8)	**0.02**
Hypertension, *n* (%)		59 (37.8)	41 (44.1)	18 (28.6)	0.05
Diabetes, *n* (%)		26 (16.7)	19 (20.4)	7 (11.1)	0.13
Kidney disease, *n* (%)		4 (2.6)	2 (2.2)	2 (3.2)	1.00
Cancer, *n* (%)		6 (3.9)	3 (3.2)	3 (4.8)	0.69
Ischemic heart disease, *n* (%)		17 (10.9)	10 (10.8)	7 (11.1)	0.94
Number of comorbidities		1 (0–2)	1 (0–2)	1 (0–1)	0.07
Sartan, *n* (%)		25 (16.1)	20 (21.5)	5 (8.1)	0.03
ACE inhibitors, *n* (%)		13 (8.4)	10 (10.8)	3 (4.8)	0.25
Antiplatelet therapy, *n* (%)	Prophylaxis	18 (11.6)	10 (10.8)	8 (12.9)	0.45
	Anticoagulant	3 (1.9)	3 (3.2)	0	
**Emergency department**					
Hb, g/dL (*n* = 154)		13.9 (1.6)	13.7 (1.6)	14.2 (1.7)	0.05
White blood cells, ×10^6^		6.7 (5.4–9.3)	6.5 (5.4–9.3)	6.9 (5.4–9.2)	0.99
Neutrophils, %		71.9 (11.7–83.8)	69.1 (6.7–83.1)	74.6 (61.5–84.4)	**0.007**
Lymphocytes, %		10.3 (1.4–16.8)	7.1 (1.1–15.0)	12 (6.6–19.4)	**0.005**
Platelets, ×10^9^		214 (171.5–160.5)	219 (179–280)	207 (157–250)	0.15
I.N.R.		1.2 (1.1–1.3)	1.2 (0.1–1.3)	1.2 (1.1–1.3)	0.53
Aspartate transaminase, U/L		48 (36–71)	47.0 (35.0–68.0)	52.5 (42.0–76.5)	0.09
Bilirubin, mg/dL		1.17 (0.56–1.19)	1.19 (0.62–1.19)	0.77 (0.50–1.19)	0.05
Median (IQR) serum creatinine		0.9 (0.7–1.0)	0.8 (0.7–1.0)	0.9 (0.8–1.1)	0.05
Lactate Dehydrogenase, U/L		351.5 (288–458)	341 (288–436)	391 (286–599)	0.15
D-dimer, FEU mg/L		421.5 (279–971)	477 (307–2078)	394 (241–571)	0.08
Temperature, °C		37.5 (1.0)	37.3 (1.0)	37.8 (1.1)	**0.007**
Systolic arterial pressure, mmHg		130 (120–140)	130 (120–140)	130 (120–140)	**0.04**
Heart rate, bpm		91.9 (15.6)	89.7 (16.2)	95.1 (14.1)	**0.03**
Respiratory rate, bpm		24 (22.0–27.5)	24 (22–27)	24 (22–28)	0.38
pH		7.47 (7.44–7.49)	7.47 (7.44–7.50)	7.47 (7.45–7.49)	0.87
PaCO_2_, mmHg		33 (30.0–38.0)	33.3 (30.0–39.9)	33 (30.3–35.3)	0.30
PaO_2_, mmHg		67.9 (59–82)	69.7 (60.3–90.0)	61.8 (57–73)	**0.005**
PaO_2_/FiO_2_, mmHg		269 (168–310)	267 (169–312)	271 (151–295)	0.52
**Outcomes**					
Duration of CPAP, days		4 (2–7)	6 (4–9)	2 (1–4)	**<0.001**
Hospital length of stay, days		24 (17–32)	23 (17–30)	25.5 (17–38)	0.41
In-hospital mortality, *n* (%)		30 (19.2)	0	27 (42.9)	**<0.001**

Data are reported as means (standard deviation) or medians (interquartile range), as appropriate. Statistically significant comparisons are marked in bold. ACE = angiotensin converting enzyme; Hb = hemoglobin; PaO_2_ = partial pressure of oxygen; PaCO_2_ = partial pressure of carbon dioxide; FiO_2_ = Inspired oxy-gen fraction.

**Table 2:** Clinical characteristics and outcomes in patients that failed CPAP and were intubated or undergone a NIV trial.

	CPAP + ETI (*n* = 18)	CPAP + NIV (*n* = 45)	*p*-Value
Males, *n* (%)	15 (83.3)	39 (86.7)	0.73
Age, years	60.5 (58–65)	58 (55–64)	0.14
Days from symptoms onset to hospital admission, *n*	8 (6–11)	8 (6–10)	0.99
**Comorbidities**			
Previous respiratory disease, *n* (%)	2 (11.1)	3 (6.7)	0.62
Smoke, *n* (%)	1 (5.9)	2 (4.4)	1.00
Hypertension, *n* (%)	9 (50.0)	9 (20.0)	**0.02**
Diabetes, *n* (%)	2 (11.1)	5 (11.0)	1.00
Ischemic Heart disease, *n* (%)	3 (16.7)	4 (8.9)	0.40
Sartan, *n* (%)	3 (17.7)	2 (4.4)	0.12
ACE inhibitors, *n* (%)	2 (11.8)	1 (2.2)	0.18
**Emergency department**			
White blood cells, ×10^6^	8.5 (6.1–10.3)	6.8 (4.9–8.5)	0.12
Neutrophils, %	14.0 (8.9–82.5)	79.6 (68.3–84.5)	**0.02**
Lymphocytes, %	1.4 (0.6–11.3)	14.5 (9.5–22.3)	**<0.001**
Platelets, ×10^9^	222 (154.0–337.5)	205 (157–248)	0.31
I.N.R.	1.4 (1.2–1.6)	1.2 (1.1–1.2)	**<0.001**
Bilirubin, mg/dL	1.19 (0.99–1.70)	0.66 (0.41–0.97)	**0.001**
Serum creatinine, mg/dL	1 (0.7–1.2)	0.9 (0.8–1.0)	0.61
Lactate Dehydrogenase, U/L	602 (430–725)	324.5 (252.5–451.0)	**0.005**
D-dimer, mg/L FEU	2886 (414–20,333)	319 (212–520)	0.08
Respiratory rate, bpm	25 (24–30)	24 (22–28)	0.43
pH	7.48 (7.46–7.51)	7.47 (7.43–7.49)	0.08
PaCO_2_, mmHg	35.5 (33–40)	32.1 (28.9–35.0)	**0.002**
PaO_2_, mmHg	62.5 (56–73)	61.8 (58.2–72.6)	0.51
PaO_2_/FiO_2_, mmHg	151 (91–267)	281 (209.5–321.0)	**0.005**
**At CPAP failure**			
Duration of CPAP, days	2.5 (2–7)	2 (1–3)	0.05
Respiratory rate, bpm	34.7 (9.0)	25.2 (6.5)	**<0.001**
pH	7.46 (0.04)	7.45 (0.04)	0.27
PaCO_2_, mmHg	39 (4.5)	38.4 (5.3)	0.68
PaO_2_, mmHg	67 (58–83)	99 (83.5–127.5)	**<0.001**
PaO_2_/FiO_2_, mmHg	99 (82–112)	143 (121–199)	**<0.001**
Lymphocytes, %	5.5 (3.0–6.6)	10.4 (4.4–18.0)	0.05
Platelets, ×10^9^	270.6 (105.5)	282.1 (99.0)	0.95
I.N.R.	1.3 (1.2–1.7)	1.2 (1.1–1.3)	0.36
Bilirubin, mg/dL	1.19 (0.97–1.28)	1.02 (0.45–1.66)	0.48
Serum creatinine, mg/dL	0.7 (0.6–0.8)	0.7 (0.7–0.9)	0.58
D-dimer, mg/L FEU	1577 (381–5200)	348 (259–567)	0.10
**Outcomes**			
Length of hospital stay, days	19 (15–24)	29 (18–39)	0.05
In-hospital mortality, *n* (%)	14 (77.8)	13 (28.9)	**0.001**

Data are reported as means (standard deviation) or medians (interquartile range), as appropriate. Statistically significant comparisons are marked in bold. ACE = angiotensin converting enzyme; Hb = hemoglobin; PaO_2_ = partial pressure of oxygen; PaCO_2_ = partial pressure of carbon dioxide; FiO_2_ = inspired oxygen fraction.

**Table 3:** Clinical characteristics and outcomes of patients that failed CPAP + NIV treatment and were intubated vs. patients that succeed CPAP + NIV at time of hospital admission and at the time of failure.

	NIV Success (*n* = 16)	NIV Failure (*n* = 29)	*p*-Value
Males, *n* (%)	12 (75.0)	27 (93.1)	0.09
Age, years	61 (55.5–65.5)	57 (51–63)	0.34
Days from symptoms onset to hospital admission	8 (8–11)	8 (5.5–9.0)	0.23
Duration of CPAP, days	2 (1.0–3.5)	2 (1–3)	0.62
**Comorbidities**			
Previous respiratory disease, *n* (%)	1 (6.3)	2 (6.9)	1.00
Smoke, *n* (%)	1 (6.3)	1 (3.5)	1.00
Hypertension, *n* (%)	3 (18.8)	6 (20.7)	1.00
Diabetes, *n* (%)	3 (18.8)	2 (6.9)	0.33
Ischemic Heart disease, *n* (%)	2 (12.5)	2 (6.9)	0.61
Sartan, *n* (%)	1 (6.3)	1 (3.6)	1.00
ACE inhibitors, *n* (%)	1 (6.3)	0 (0.0)	0.36
**Emergency department**			
Mean (SD) Hb, g/dL	14.0 (1.8)	14.7 (1.5)	0.21
White blood cells, ×10^6^	6.7 (3.9–8.4)	7.0 (5.2–8.5)	0.39
Neutrophils, %	82.3 (61.9–84.9)	76.9 (70.2–83.8)	0.90
Lymphocytes, %	14.8 (9.6–29.6)	14.2 (9.5–20.8)	0.60
Platelets, ×10^9^	210.5 (165.5–234.5)	190 (156–249)	0.75
I.N.R.	1.2 (1.1–1.2)	1.1 (1.1–1.2)	0.15
Bilirubin, mg/dL	0.39 (0.33–0.62)	0.73 (0.47–1.02)	0.12
Serum creatinine, mg/dL	0.8 (0.6–1.2)	1 (0.9–1.0)	0.12
Lactate dehydrogenase, U/L	364.5 (293–402)	316 (236–458)	0.52
D-dimer, mg/L FEU	404.5 (279–609)	314 (186–468)	0.50
Temperature, °C	37.5 (1.2)	37.9 (1.0)	0.31
Systolic arterial pressure, mmHg	125 (118–130)	140 (125–150)	**<0.001**
Mean arterial pressure, mm/g	93.4 (10.8)	99 (14.1)	0.17
Heart rate, bpm	94.1 (13.3)	99.2 (15.7)	0.28
Respiratory rate, bpm	24 (23.5–24.5)	24 (22–30)	0.42
pH	7.46 (7.40–7.48)	7.47 (7.44–7.49)	0.28
PaCO_2_, mmHg	32.3 (30–35)	32 (28–35)	0.74
PaO_2_, mmHg	64.5 (59–73)	61.8 (55.9–72.6)	0.71
PaO_2_/FiO_2_, mmHg	281 (271.0–326.5)	280 (196–317)	0.64
**Outcomes**			
Length of hospital stay, days	28.5 (24.5–36.5)	29 (17–39)	0.71
In hospital mortality, *n* (%)	0 (0.0)	13 (44.8)	**<0.001**

Data are reported as means (standard deviation) or medians (interquartile range), as appropriate. Statistically significant comparisons are marked in bold. ACE = angiotensin converting enzyme; Hb = hemoglobin; PaO_2_ = partial pressure of oxygen; PaCO_2_ = partial pressure of carbon dioxide; FiO_2_ = inspired oxygen fraction.

**Table 4:** Characteristics at admission and at failure time of patients that failed CPAP + NIV treatment and were intubated vs. patients that succeeded CPAP + NIV.

	CPAP + ETI (*n* = 18)	CPAP + NIV + ETI (*n* = 29)	*p*-Value
Males, *n* (%)	15 (83.3)	27 (93.1)	0.279
Age, years	60.5 (57.7–65)	57 (50.5–63.5)	0.047
Days from symptoms onset to hospital admission	8 (6–11)	8 (5–9)	0.722
**Comorbidities**			
Previous respiratory disease, *n* (%)	2 (11.1)	2 (6.9)	0.498
Smoke, *n* (%)	1 (5.9)	1 (3.4)	0.608
Hypertension, *n* (%)	9 (50.0)	6 (20.7)	0.039
Diabetes, *n* (%)	2 (11.1)	2 (6.9)	0.498
Ischemic heart disease, *n* (%)	3 (16.7)	2 (6.9)	0.279
Sartan, *n* (%)	3 (17.6)	1 (3.4)	0.135
ACE inhibitors, *n* (%)	2 (11.8)	0 (0)	0.131
**Emergency department**			
White blood cells, ×10^6^	9.2 (3.1)	7.2 (2.6)	0.106
Neutrophils, %	85.2 (82.5–90.5)	76.9 (69.7–83.8)	**0.003**
Lymphocytes, %	8.5 (5.4–12.5)	14.2 (9.1–21.2)	**0.008**
Platelets, ×10^9^	271.0 (159.9)	201.2 (58.9)	**0.039**
I.N.R.	1.4 (1.2–1.6)	1.1 (1.1–1.2)	**<0.001**
Bilirubin, mg/dL	1.19 (0.91–1.84)	0.73 (0.47–1.02)	**0.005**
Serum creatinine, mg/dL	1.0 (0.7–1.2)	1.0 (0.9–1.1)	0.963
Lactate dehydrogenase, U/L	602 (430–725)	316 (233–465)	**0.001**
D-dimer, mg/L FEU	2885 (334–27,899)	314 (186–468)	0.062
Respiratory rate, bpm	25.0 (23–30)	24.0 (22–30)	0.674
pH	7.48 (7.46–7.51)	7.47 (7.44–7.49)	0.192
PaCO_2_, mm/Hg	35.5 (4.6)	31.8 (4.9)	0.053
PaO_2_/FiO_2_, mm/Hg	173 (89–268)	253 (190–319)	**0.003**
**At CPAP or NIV failure**			
Lymphocytes, %	5.5 (3.0–6.6)	8.2 (6.1–14.0)	**0.03**
Platelets, ×10^9^	270.6 (105.5)	318.2 (124.4)	0.29
I.N.R.	1.3 (1.2–1.7)	1.2 (1.1–1.3)	0.15
Bilirubin, mg/dL	1.19 (0.97–1.28)	0.76 (0.46–1.03)	0.05
Serum creatinine, mg/dL	0.7 (0.6–0.8)	0.7 (0.6–0.8)	0.84
D-dimer, mg/L FEU	1577 (381–5200)	826 (276–2570)	0.52
Respiratory rate, bpm	33.5 (24–42)	29.5 (26–30)	0.06
pH	7.46 (0.04)	7.48 (0.04)	0.19
PaCO_2_, mmHg	39 (4.5)	36.8 (5.0)	0.15
PaO_2_/FiO_2_, mmHg	99 (82–112)	110 (86–150)	0.34
**Outcomes**			
Length of hospital stay, days	19 (14.5–25.0)	27.8 (17–39)	0.162
In hospital mortality, *n* (%)	14 (77.8)	13 (44.8)	**0.026**

Data are reported as means (standard deviation) or medians (interquartile range), as appropriate. Statistically significant comparisons are marked in bold. ACE = angiotensin converting enzyme; Hb = hemoglobin; PaO_2_ = partial pressure of oxygen; PaCO_2_ = partial pressure of carbon dioxide; FiO_2_ = inspired oxygen fraction.

## Data Availability

D.C. had full access to all the data in the study and take responsibility for the integrity of the data and the accuracy of the data analysis and had final responsibility for the decision to submit for publication. The anonymized datasets used and analyzed during the current study are available from the corresponding author on reasonable request.
